# Quantifying target-specific motion in anal cancer patients treated with intensity modulated radiotherapy (IMRT)

**DOI:** 10.1016/j.radonc.2016.08.011

**Published:** 2016-10

**Authors:** Lisa Durrant, Maxwell Robinson, Maria A. Hawkins, Frank Van den Heuvel, Rebecca Muirhead

**Affiliations:** CRUK/MRC Oxford Institute for Radiation Oncology, University of Oxford, UK

**Keywords:** Anal cancer IMRT, Inguinal node, Elective irradiation, Margin reduction

## Abstract

**Background and purpose:**

Intensity modulated radiotherapy requires all target areas to be treated by a single radiotherapy plan. In anal cancer, the pelvic nodes, inguinal nodes and primary tumour represent three different targets. We aim to calculate target-specific motion in anal cancer radiotherapy, when delivered using a single pelvic online auto-match.

**Materials and methods:**

Twenty consecutive patients treated using IMRT at a single institution were studied. CBCTs were retrospectively re-matched around the inguinal nodes and primary tumour. Match values were recorded relative to origin, defined as pelvic CBCT auto-match. Systematic and random errors were quantified to determine target-specific motion and suggested margins calculated using van Herk formulae.

**Results:**

The suggested margins to cover the independent motion of the inguinal and anal targets for LR, CC and AP set up around the inguinal nodes were 1.5 mm, 2.7 mm and 2.8 mm; and the primary tumour were, 4.6 mm, 8.9 mm and 5.2 mm respectively.

**Conclusions:**

Target-specific set up will likely result in reduced treatment volumes and as such reduced toxicity. This is the first time a relationship has been described between pelvic bones, inguinal nodes and primary tumour. The PLATO study will prospectively assess the toxicity and outcomes of this target-specific margins strategy.

Radical chemoradiotherapy (CRT) is the standard treatment for squamous cell carcinoma of the anal canal, achieving local control rates of 73% at 3 years [1], [2]. Squamous cell carcinoma of the anus is a loco-regional disease with spread into the regional pelvic and inguinal nodes. Only <13% of patients present with distant disease [3]. As such, during CRT, the primary tumour and involved nodes receive a tumoricidal dose of radiation while the remaining regional pelvic and inguinal lymph nodes receive an elective dose with the aim of eliminating microscopic disease.

Intensity modulated radiotherapy (IMRT) is now used to conform the radiotherapy plan around target volumes resulting in reduced toxicity [4]. IMRT is delivered using a simultaneous integrated boost (SIB) to cover the primary tumour, involved nodes and the elective nodes within the same inverse planned IMRT delivery. The elective nodes include the pelvic nodes, namely internal and external iliac, obturators, presacral and lower section of mesorectal nodes, and the inguinal nodes.

In IMRT, the gross tumour volume (GTV), clinical target volume (CTV), and planned target volume (PTV) are created in accordance with ICRU 50, 62 and 83 [5], [6], [7]. The CTV to PTV margin is often referred to as the “set up margin” and incorporates a number of potential errors, the largest being internal target motion of the CTV over the 5½ week of radiotherapy [8], [9]. In anal cancer radiotherapy, there are 3 geographically distinct targets: the pelvic lymph nodes, inguinal lymph nodes and the primary anal tumour or gross tumour volume (GTV_A). Due to the differing surrounding muscle groups and organs, we hypothesise these three targets have varying degrees of motion and as such require individualised CTV to PTV margins. The defining of margins for different targets has already been investigated in prostate, cervix and bladder cancer where the primary tumour and elective nodal groups move independently [10], [11], [12], [13].

Chen et al. investigated margins in anal cancer. They used a 5 mm margin from CTV to PTV and a bony pelvis auto-match on a Megavolt Cone Bean CT (MVCBCT) for verification. They analysed whether further margins were required with and without the use of daily online imaging, suggesting that with the use of daily imaging a 5 mm margin was adequate [14]. As CBCT and kilo Voltage (kV) imaging are comparable in calculating shifts based on pelvic matches, this study would suggest that with 5 mm margin, the elective pelvic nodal volume is adequately covered using daily online kV or CBCT match. However the target-specific motion of the inguinal nodes or anus, while using a pelvic auto-match was not assessed or incorporated, into the margin calculation.

The issue with a standard set up margin around all three anal cancer targets is the potential for either geographical miss in areas where there is increased motion; or increased toxicity where the margin which covers the most mobile target being unnecessarily large for the more static targets. Chen et al. confirmed that the smaller margins reduced the doses to organs at risk [14]. Robinson et al. also reported a similar finding with reduced bone marrow toxicity using smaller margins [15]. Radiation to these different targets results in different toxicities. Whole pelvis versus prostate alone studies have demonstrated that irradiation of pelvic nodes results in increased gastrointestinal toxicity [16], [17]. Treatment of inguinal nodes is associated with dermatitis, groin pain and lymphoedema of the legs [18]. In addition, when the set up margin is added to create the PTV, the inguinal volumes increase the dose to the genitalia causing toxicities such as vaginal stricturing, fistulas, dryness and dyspareunia [19]. Lastly the major toxicity of anal irradiation is ulceration and faecal incontinence [20]. As such it is important to use accurate, target-specific margins to minimise each of these associated toxicities.

This study aims to quantify the target-specific CTV to PTV growth to aid in the decision process in determining adequate margins required in the elective inguinal nodes and primary anal tumour when using an online CBCT image guided radiotherapy (IGRT) protocol.

## Materials and methods

### Patient selection

Twenty consecutive anal cancer patients, treated between 2013 and 2015, at Oxford University Trust; were evaluated within this retrospective study. Patients had a diagnosis of squamous cell carcinoma; had completed full dose chemoradiotherapy to primary tumour and all elective nodes according to United Kingdom (UK) IMRT guidelines [21], [22]; did not have a hip prostheses; and had uninvolved inguinal nodes as determined radiographically by FDG_PET and MRI imaging.

### Treatment

Patients were immobilised supine with a comfortably full bladder, indexed knee support under the popliteal fossa and ankles stocks. Treatment was delivered over 28 fractions. Volumes were delineated as per UK guidance [21], [22] by an experienced clinician on a 2.5 mm contrast enhanced planning CT. In summary the primary gross anal tumour (GTV_Anal) was delineated and enlarged by 25 mm to create PTV_Anal (15 mm CTV and 10 mm for PTV) which received 50.4 or 53.2 Gy according to stage. The involved pelvic nodes were delineated plus a margin of 20 mm to create PTV_Nodes and treated to a dose of 50.4 Gy. Lastly the elective node volume (CTV_Elective) was delineated and enlarged by 10 mm to create PTV_Elective which received 40 Gy. Both elective nodal targets were delineated or edited to exclude local muscles and bones. Treatments were inversely planned using 7–9 field IMRT with a simultaneous integrated boost.

Chemotherapy was delivered with Mitomycin C 12 mg/m^2^ on day 1 and Capecitabine 825 mg/m^2^ twice daily on all days of radiotherapy.

Patients underwent daily imaging using a Varian Clinac IX with OBI (CBCT) (Varian Medical Solutions, CA, USA). On fractions 1–5, 10, 15, 20, 25 a CBCT was performed with paired orthogonal kV imaging all other fractions. Online pelvic auto-match was performed daily. Initially an auto-match including pitch, yaw and roll rotation was performed. If rotation was <3 mm a further pelvic auto-match was performed without rotation, prior to shifts and treatment. The patient was repositioned and re-imaged if rotation was >3 degrees.

### Retrospective image matching

Twenty patients with 9 CBCTs each were analysed. The 180 CBCTs underwent 3 matches (pelvic, inguinal, and anal) summating 540 matches.

For matching purposes CTV_Elective was modified to create one further volume of interest (VOI), the inguinal node volume (VOI_Inguinal) encompassing the inguinal nodes alone. The GTV_Anal was used as the anal VOI (VOI_Anal). The different volumes of interest are illustrated in Fig. 1.

Three matches were performed to represent our departmental standard range of corrections; lateral or left/right (LR), longitudinal or cranial/caudal (CC) and vertical or anterior/posterior (AP) were permitted. Rotations were not measured, nor corrected.

(1) A rigid, pelvic auto-match using a region of interest (ROI) to encompass the bony pelvis. ROI borders were: the anterior border of the symphysis pubis, anterior border the sacrum, femoral heads laterally and the full extent of the CBCT image (16 cm on a Varian Clinac IX). The values for the pelvic match were recorded to act as a point of origin and reference for further matches.
(2) A rigid, auto-match to VOI_Anal.
(3) A rigid, auto-match to VOI_Inguinal.

Matches were assessed visually by an experienced radiographer to ensure clinical relevance and analysis pooled by combining the measurements from all 9 CBCT images for each patient.

All matches used the registration module of Eclipse Treatment Planning System Version 13 (Varian Medical Systems, CA, USA).

### Systemic and random motion calculations

We are interested in the inter-fractional variation of the position of the VOI under investigation with respect to the bony anatomy. The use of the word “motion” indicates this variation in the remainder of the text.

To calculate motion for the inguinal nodes and primary tumour relative to the pelvic bones; the shift from the pelvic auto-match to the VOI_Inguinal and VOI_Anal respectively was calculated. The systematic (*Σ*) and random motion (*σ*) was calculated for both areas for all 3 degrees of freedom as per Van Herk et al. publication, and which is reflected in the IPEM guidance document [23], [24].

### Target-specific margin calculations

Van Herk et al. equation (2.5*Σ* + 0.7*σ*) was used to propose target-specific motion for inguinal nodes and primary tumour [24]. While this equation is routinely used to calculate set up margins, we have used it in this case to quantify the motion in order to guide the determination of a PTV margin for each target.

### Reproducibility analysis

To ensure reproducibility and consistency of matches to VOI_Inguinal and VOI_Anal, the auto-matches for day 2 CBCT, in all 20 patients were repeated a further 2 times, by the same observer, summating 120 matches. The standard deviation (SD) of these 3 auto-matches was then calculated.

The data were then pooled by calculating a mean from each match in each direction for both inguinal and primary tumour targets.

## Results

Patient and tumour demographics are documented in Table 1.

387 of 540 (85.8%) of individual VOI_Inguinal matches were equal or smaller than the comparably matched VOI_Anal match relative to the pelvic auto-match. The range of individual inguinal node matches (mm) were −2.4 to 2.2, −8.5 to 7.3, −4.6 to 6.4 in the LR, CC and AP directions respectively; and −9.8 to 4.2, −13.3 to 17.2, −6.9 to 12.7 respectively in the primary tumour matches. Fig. 2 illustrates a patient were the pelvic match aligns well with VOI_Inguinal but poorly with VOI_Anal.

The pooled analysis for each patient demonstrated that inguinal node matches conformed more closely to the pelvic auto-match than the primary tumour in all directions (Fig. 3).

The differences between the shifts between the inguinal nodes and the primary target demonstrate while there is more correlation between the shifts in the lateral direction, the two targets move independently in terms of both distance and direction to each other (Fig. 4).

### Systematic and random errors

In all cases the systematic and random errors were larger for the primary tumour than the inguinal nodes, they are documented in Table 2.

### Target-specific set up calculations

The suggested margins, to incorporate the target-specific motion for LR, CC and AP directions around the VOI_Inguinal were 2 mm, 3 mm and 3 mm respectively. Around VOI_Anal the margin required to encompass the increased motion of this target were 5 mm, 9 mm and 5 mm respectively.

### Reproducibility of matches

The mean (SD) of all the standard deviations for each match in LR, CC and AP directions was 0.44 mm (0.31), 0.88 mm (0.95) and 0.75 mm (0.71) respectively in the inguinal node matching and 0.42 mm (0.28); 1.18 mm (0.87), 0.73 mm (0.61) respectively in the primary tumour match. The majority of deviations is sub-millimetre and confirms the auto-match process is reliable.

**Figure 5:**
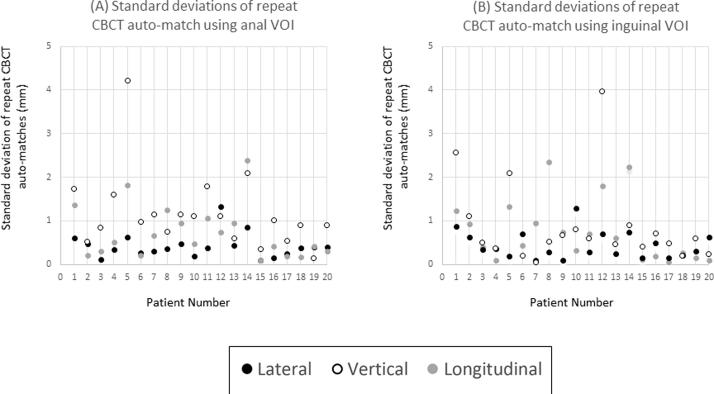
Scatter plots illustrating the standard deviation of 3 repeat auto-matches around the (A) anus VOI and (B) inguinal VOI.

**Fig. 1:**
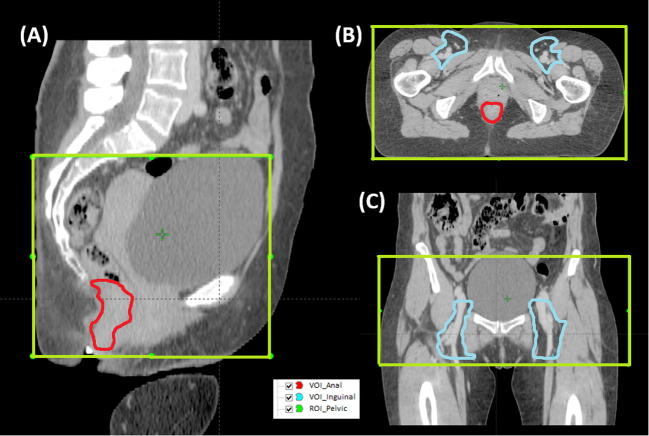
Planning CT scan demonstrating the VOI used for the primary tumour (VOI_Anal, red contour) and the inguinal nodes (VOI_Elective, light blue contour) and the ROI used for pelvic auto-match (green contour) in (A) Sagittal, (B) Axial and (C) Coronal slices.

**Fig. 2:**
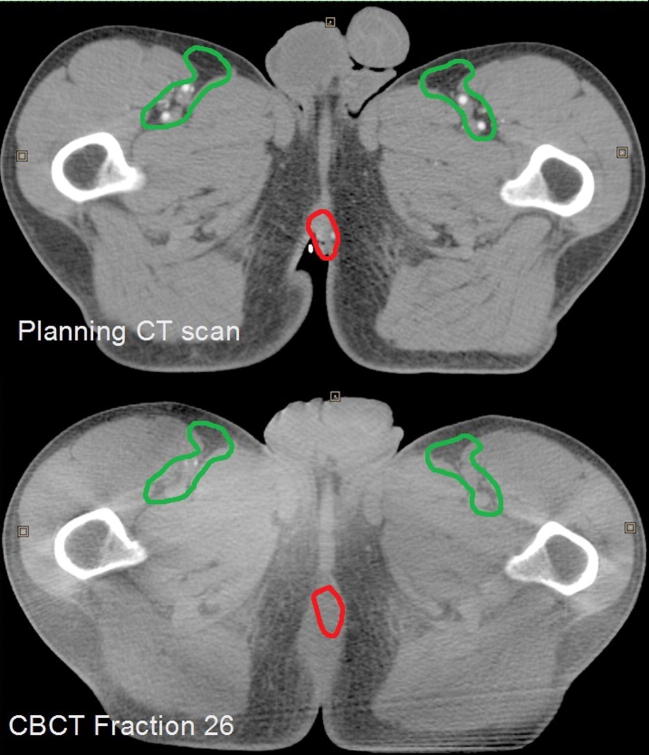
Patient where the CBCT pelvic bony auto-match resulted in good alignment of the inguinal target (green contour) but poor alignment of anal target (red contour). (For interpretation of the references to colour in this figure legend, the reader is referred to the web version of this article.)

**Fig. 3:**
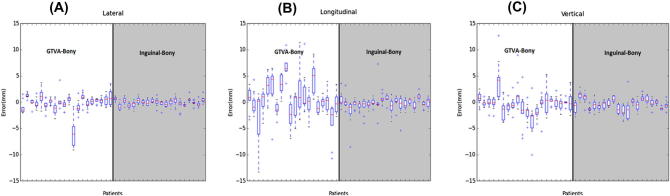
VOI_Anal (grey area) and VOI_Inguinal (white area) auto-matches for each patient in 3 degrees of freedom. Red line indicates median; box represents the 25th and 75th centiles; whiskers the minimum and maximum measured values. (For interpretation of the references to colour in this figure legend, the reader is referred to the web version of this article.)

**Fig. 4:**
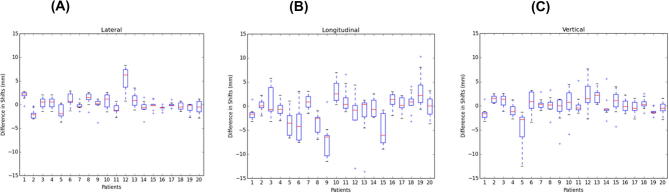
Difference in motion between the inguinal node and primary tumour targets in lateral, longitudinal and vertical directions.

The standard deviation of the 3 repeated matches in each direction for the two targets is shown in Supplementary material Fig. 5.

## Discussion

To our knowledge the concept and quantification of target-specific motion has not been described previously in anal cancer. In this study we have quantified the motion around the inguinal nodes and primary tumour, and offer appropriate margins to ensure adequate coverage and reduce un-necessary toxicity; which can be prospectively verified.

In the clinical application of these data, it must be acknowledged that standard isotropic margins are often chosen for ease of use. In addition, although the motion of the CTV over the treatment course is the predominant factor in CTV to PTV margins, there are other potential errors for example interclinician variation in delineation, table sag, laser calibration, etc. Finally, the accuracy of different linear accelerators must be considered; when considering margins <3 mm it must be acknowledged that some linear accelerators do not have this degree of accuracy in delivery and slice thickness of some scanners may exceed the set up. As such we would suggest the use of 10 mm from CTV_Anal to PTV_Anal as a pragmatic compromise. It must also be taken into account that the GTV_Anal was used as the VOI_Anal, therefore an additional margin from GTV_Anal to CTV_Anal must be used for microscopic disease prior to adding the set up margin. Similarly the pragmatic margin for CTV_Inguinal to PTV_Inguinal would be 5 mm.

Our study did not investigate rotational moves however the three degrees of freedom investigated, represent the online corrections performed on the majority of standard linear accelerators and therefore our study is appropriate for the purposes of calculating set up for routine clinical care.

It must be noted that the matches and as such the errors and margins calculated are only relevant for similar verification techniques, ROIs / VOI, threshold for shifts and matches. For centres using different delineation techniques for their elective inguinal nodes, or imaging/matching techniques or frequencies, or have a minimum threshold for shifting; a centre specific margin should be calculated. In addition, individual centres not involved in large scale audits or trials should intermittently prospectively assess these margins and outcomes to ensure they are unchanged by slight changes in matches or delineation, by changes in staff or equipment.

We selected patients without involved inguinal nodes. The authors had concerns that the soft tissue auto-match may have been improved by the incorporation in the VOI of a defined large node. By selecting patients without involved nodes the calculation of set up is performed without the benefit of defined lymph nodes and as such would be appropriate for all anal cancer patients with and without nodes as this margin is required around elective nodes even in those patients that are node positive.

Further patient specific patient individualisation may be possible for outliers who require bigger margins. If these patients can be identified prior to treatment, appropriate margins can be used from outset. Factors that could affect the required margins include patient sex, BMI, tumour size, location, bowel and bladder filling at outset. Alternatively a number of adaptive planning techniques have been suggested such as the use of a “multiple adaptive plan” where multiple plans are created at outset and a plan of the day is selected, the use of an adaptive morphing algorithm or daily plan adjustment following online image assessment [25], [26], [27]. Anecdotally the image quality, bowel gas and tumour shrinkage, were factors that affected the anal match.

Previously the UK IMRT guidance suggested 10 mm CTV to PTV margin around the inguinal nodes which our study suggests could be reduced to 5 mm. The Anal GTV to CTV and CTV to PTV margins were previously combined in the UK IMRT guidance suggesting a total margin of 25 mm. Data from skin squamous cell carcinoma would suggest 11 mm in early disease and 14 mm in locally advanced disease is an appropriate margin for microscopic disease [28]. Therefore in early disease 20 mm GTV_Anal to PTV_Anal (GTV_Anal to CTV_Anal = 10 mm + CTV_Anal to PTV_Anal = 10 mm) and in locally advanced disease suggested GTV_Anal to PTV_Anal margin of 25 mm (GTV_Anal to CTV_Anal = 15 mm + CTV_Anal to PTV_Anal = 10 mm).

In terms of margins used internationally; the RTOG 0529 protocol suggested a 1 cm CTV to PTV margin [29] while the TROG atlas recognise the different verification protocols may allow different margins, suggesting 5–7 mm with daily online imaging and 10 mm without [30]. The numerous series published used a wide range of CTV to PTV margins ranging from 5 mm to 15 mm [31], [32], [33], [34] but none of the published literature suggests individualised margins for different targets. This widespread variation suggests there remains uncertainty regarding the correct margin and the need for studies similar to our own.

While the quantification of margins is essential in anal radiotherapy, other potential errors in the move to IMRT must be addressed. Delineation and planning discrepancies are problematic in the move from conformal treatment to IMRT in anal cancer. RTOG 0529 had a thorough quality assurance programme and reported 33% had incorrectly delineated inguinal node compartments. 81% had unacceptable plans initially, despite clear delineation and planning instructions and an atlas; and 46% had continued unacceptable plans following feedback. As such it is important to audit and report outcomes of large series after implementation such as the national UK audit of IMRT in anal cancer [35].

Chen et al. already reported reductions in standard margins across different target areas reduced the dose to organs at risk [14]. Although dosimetric modelling of consequences to organs at risk using these target-specific margins could be performed, the prospective assessment of toxicity and outcomes within a large international trial is far superior method of assessment. The upcoming PLATO study (ISRCTN88455282), investigating individualised radiotherapy doses, plans to incorporate these margins and has a built in safety and toxicity pilot which will assess these.

## Conflicts of interest statement

None to declare.

## Figures and Tables

**Table 1:** Patient and tumour characteristics.

	All patients (*n* = 20)
Age (y), median (range)	54 (39–73)

Gender	
Male	5
Female	15

T stage	
T1	5
T2	11
T3	3
T4	1

N stage	
N0	15
N1	4
N2	0
N3	1

RT dose to primary tumour (Gy)	
50.4 Gy	12
53.2 Gy	8

GTV_A Volume (cc’s), median (range)	33.6 (3–175)

*Abbreviations:* BMI = RT, radiotherapy; GTV, gross tumour volume.

**Table 2:** Patient and Tumor characteristics.

	Lateral (cm)	Longitudinal (cm)	Vertical (cm)	Rotational (cm)
VOI_Anal				
*M*_population_	−0.2	0.7	−0.3	0
*Σ*_population_	1.5	2.7	1.5	0
*σ*_population_	1.1	2.8	1.8	0
*σ*_population_RMS	1.2	3	2	0

VOI_Inguinal				
*M*_population_	0	−0.2	−0.3	0
*Σ*_population_	0.4	0.7	0.9	0
*σ*_population_	0.6	1.2	0.8	0
*σ*_population_RMS	0.6	1.4	0.9	0

*Abbreviations: M*_population_ = overall population mean set-up error; *Σ*_population_ = population systematic set-up error; *σ*_population_ = random set-up error; *σ*_population_RMS = population random error using root mean square.
